# Supplementary material for: Graphitized Carbon Xerogels for Lithium-Ion Batteries
Source: Materials (Basel). 2019 Dec 26;13(1):119. doi: 10.3390/ma13010119 (PMC6981815; doi:10.3390/ma13010119)
Supplement: Supplementary file 1 [file materials-13-00119-s001.pdf]

materials

Supplementary Materials

Graphitized Carbon Xerogels for Lithium-ion Batteries

Maria Canal-Rodríguez, Ana Arenillas *, Sara F. Villanueva, Miguel A. Montes-Morán, and J. Angel Menénedez *

Instituto Nacional del Carbón (INCAR-CSIC), Francisco Pintado Fe 26, 33011 Oviedo, Asturias, Spain; mcanal@leitat.org (M.C.-R); s.villanueva@incar.csic.es (S.F.V.); miguel@incar.csic.es (M.A.M.-M.)

* Correspondence: aapuente@incar.csic.es (A.A.); angelmd@incar.csic.es (J.A.M.)

Received: 27 November 2019; Accepted: 20 December 2019; Published: date

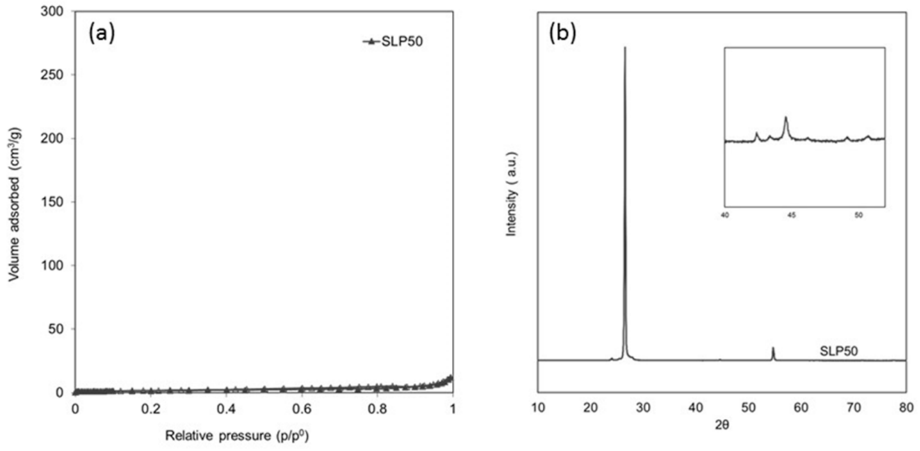

Figure S1. N_2 adsorption-desorption isotherm (**a**) and XRD pattern (**b**) of the reference material.

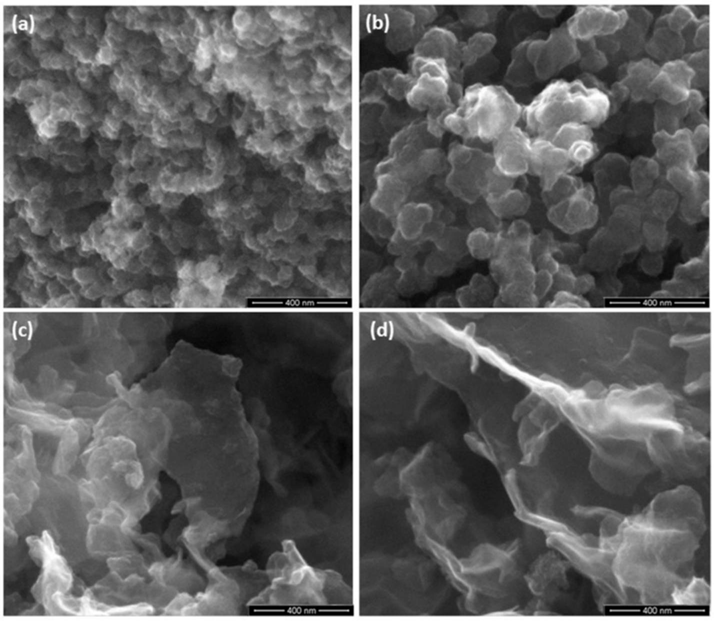

Figure S2. SEM images of: (**a**) GX-100, (**b**) GX-300, (**c**) GX-100GO and (**d**) GX-300GO.

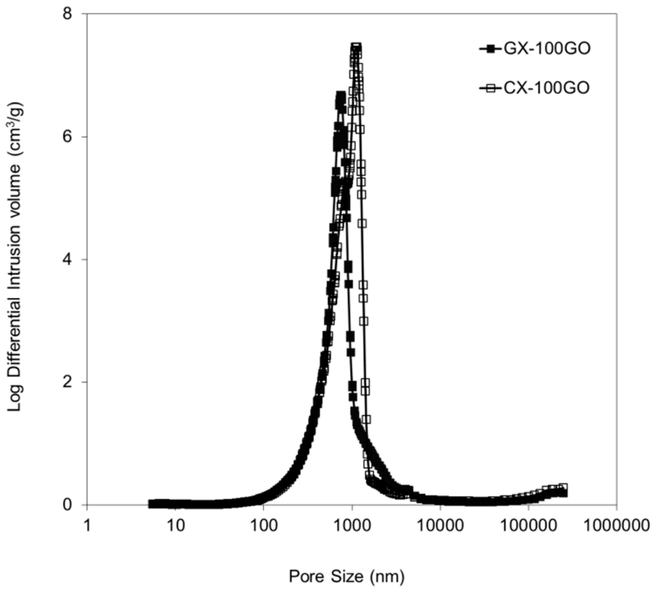

Figure S3. Pore size distributions obtained from mercury porosimetry of GX-100GO and its precursor carbon xerogels (CX-100GO).

© 2019 by the authors. Submitted for possible open access publication under the terms and conditions of the Creative Commons Attribution (CC BY) license (http://creativecommons.org/licenses/by/4.0/).
